# A Novel Method for the Detection of SARS-CoV-2 Based on Graphene-Impedimetric Immunosensor

**DOI:** 10.3390/ma14154230

**Published:** 2021-07-29

**Authors:** Gabriel C. Zaccariotto, Martin K. L. Silva, Giovanna S. Rocha, Ivana Cesarino

**Affiliations:** Department of Bioprocesses and Biotechnology, School of Agriculture, São Paulo State University (UNESP), Botucatu 18610-034, Brazil; g.zaccariotto@unesp.br (G.C.Z.); martin.leme@unesp.br (M.K.L.S.); giovanna.s.rocha@unesp.br (G.S.R.)

**Keywords:** electrochemical sensor, reduced graphene oxide, COVID-19, SARS-CoV-2

## Abstract

Due to the SARS-CoV-2 pandemic, there has been an increase in the search for affordable healthcare devices for mass testing and rapid diagnosis. In this context, this work described a new methodology for SARS-CoV-2 detection based on an impedimetric immunosensor developed using the advantageous immobilization of antibodies in the reduced graphene oxide (rGO). The rGO was obtained by chemical synthesis from the commercial graphene oxide (GO), and the materials were morphologically, electrochemically and visually characterized. The cyclic voltammetry (CV) and electrochemical impedance spectroscopy (EIS) techniques were used to evaluate the fabrication steps of the immunosensor. The electrochemical immunoassay was considered for SARS-CoV-2 spike protein RBD detection using a impedimetric immunosensor and redox couple ([(Fe(CN)_6_)]^3−/4−^) as a probe. The immunosensor was effectively developed and applied in the detection of SARS-CoV-2 spike protein RBD in saliva samples.

## 1. Introduction

The development of rapid tests, with significant reliability, easy applicability and low cost is essential for the context of the SARS-CoV-2 pandemic [1,2]. In Brazil, specifically, the development of these devices based on the national sample of patients appears as an essential and necessary opportunity for the development of national technology with low cost and accessibility, in order to minimize the bringing down effect on the health system. Hence, developing novel electrochemical biosensors based on the detection of antigen-antibody interactions or membrane proteins of SARS-CoV-2 appears as an adequate and accessible alternative of test devices to those based on reverse transcription-polymerase chain reaction (RT-PCR) to supply the world’s current demand [3,4,5,6].

Yakoh et al. [7] developed a paper-based electrochemical platform as a screening tool to detect SARS-CoV-2 immunoglobulins. The electrochemical sensor reached a limit of detection (LOD) of 1 ng/mL for SARS-CoV-2 antibodies; however, the detection limit of the protein antigen of SARS-CoV-2 has not yet achieved the detection level in real nasal swab specimens. Zhao et al. [8] reported an electrochemical detection technology using calixarene functionalized graphene oxide for targeting RNA of SARS-CoV-2. The super sandwich-type electrochemical sensor presented an LOD of the clinical specimen of 200 copies/mL. Raziq et al. [9] developed a MIP-based electrochemical sensor for detection of SARS-CoV-2 nucleoprotein. The resulting nucleoprotein sensor showed a detection and quantification limit of 15 fM and 50 fM, respectively. Torres et al. [10] prepared a low-cost biosensor for rapid detection of SARS-CoV-2 modified with human receptor angiotensin-converting enzyme-2. According to the authors, the miniaturized biosensor can detect SARS-CoV-2 using 10 µL of sample within 4 min. Other works [11] highlight electrochemical sensors as important tools in the analysis of COVID-19 summarizing the current state-of-the-art approaches to viral electrochemical biosensors, but these technologies have not yet been developed or are under the development phase.

Different studies focusing on graphene-based electrochemical biosensors have been developed in the past decade [12,13]. Taniselass et al. [14] conducted a review highlighting the development of graphene-biosensing devices for monitoring noncommunicable disease biomarkers. The graphene research for the effective immobilization of enzymes and the accurate detection of biomolecules was discussed in another review work [15]. Our research group used the reduced graphene oxide (rGO) to immobilize enzymes for the preparation of enzymatic biosensors to monitor glucose during the second-generation ethanol production [16], to analyze neurotransmitters in urine and plasmatic serum samples [17] and to determine pesticides in food [18]. rGO was also employed as a platform in the development of an electrochemical biosensor for detection of staphylococcal enterotoxin A in milk samples [19].

In this work, the advantageous immobilization of antibodies in rGO coupled with the sensitivity of the faradic impedimetric immunosensor model was used to develop a SARS-CoV-2 antigen diagnostic device. Electrochemical characterization by cyclic voltammetry (CV) and electrochemical impedance spectroscopy (EIS) techniques evaluated the fabrication steps of the immunosensor. The antigen-antibody binding on glassy carbon (GC)/rGO platform was successfully detected by EIS and CV contributing to advances on the SARS-CoV-2 electrochemical biosensing field.

## 2. Materials and Methods

### 2.1. Reagents and Solutions

Anti-SARS-CoV-2 Spike Glycoprotein S1 antibody (monoclonal) and Recombinant human coronavirus SARS-CoV-2 Spike Glycoprotein RBD (Active) were purchased from Abcam PLC (Cambridge, UK). Graphene Oxide and Bovine Serum Albumin (BSA—lyophilized powder) were purchased from Sigma-Aldrich (São Paulo, Brazil). Lauryl sulfate sodium salt (SDS), Sodium Tetrahydridoborate (NaBH_4_), ethyl alcohol, Monopotassium phosphate (KH_2_PO_4_), Disodium hydrogen phosphate (Na_2_HPO_4_), Potassium Hexacyanoferrate (II) and (III) (K_4_[Fe(CN)_6_)] and K_3_[Fe(CN)_6_)]) were analytical grade. Solutions and dilution steps were carried out by using ultra-pure water (resistivity ≥ 18 MΩ cm^−1^) of a PURELAB Option-Q—ELGA–VEOLIA system (São Paulo, Brazil).

### 2.2. Production of Reduced Graphene Oxide

Reduced Graphene Oxide (rGO) was produced through a chemical reduction method. In a reaction flask, 20 mg of graphene oxide (5 mL of stock solution) was mixed with 15 mL of ethyl alcohol and 16.0 mg of SDS; then, the mixture was subjected to a sonication step for 30 min (75% amplitude). An amount of 8.0 mg of NaBH_4_ added into the reaction promoted the reduction of GO functionalities. The mixture was sonicated for a further 30 min. In order to eliminate any residuals reagents and clean the nanomaterial, the rGO was centrifuged three times with ethanol pure grade. After the cleaning step, the rGO was dried (60 °C overnight) and subsequently dispersed in ultra-pure water at 1.0 mg/mL prior to the immunosensor confection.

### 2.3. SARS-CoV-2 Immunosensor Fabrication

Before the surface modification, the glassy carbon (GC) electrode was polished with alumina slurries (Al_2_O_3_) and cleaned in an ultrasonic bath with ethyl alcohol for 5 min, followed by ultra-pure water for a further 5 min. Next, an aliquot 10 µL of rGO (25 μg/mL) was pipetted on the surface of the GC electrode, dried at 50 °C and the electrode was incubated on 1 mL of EDC-NHS (5 mM and 8 mM, respectively) for 1 h at room temperature. After, 10 µL of Anti-SARS-CoV-2 Spike Glycoprotein S1 antibody solution (2.5 μg/mL) was added on the surface of the GC/rGO/EDC-NHS electrode and incubated for 1 h, followed by a blocking step with BSA (1%) for 30 min. The electrode surface was softly rinsed with a phosphate buffer solution (PBS) (0.2 mol L^−1^, pH 7.4) three times during 10 s after each incubation time. Finally, the electrode was ready to the measurements of the spike protein RBD (antigen) solution.

### 2.4. Scanning Electron Microscopy

The morphology of GO and rGO were both characterized using a scanning electron microscopy (FEG–SEM) using a model Quanta 200 (FEI Company, Hillsboro, OR, USA) localized in the Electron Microscope Center of the Institute of Biosciences of Botucatu, UNESP (CME-IBB-UNESP).

### 2.5. Electrochemical Measurements

Electrochemical experiments took place in a PGSTAT-128N Autolab (Metrohm) potentiostat equipped with NOVA2.1.4 software, and the electrodes were set as follow: a glassy carbon (GC) as a working electrode, a platinum plate as an auxiliary electrode and Ag/AgCl (3.0 mol L^−1^) as the reference electrode. Cyclic Voltammetry (CV) and Electrochemical Impedance (EIS) were carried out in a 0.2 mol L^−1^ PBS (pH 7.4) solution having 0.1 mol L^−1^ of KCl and 5.0 mmol L^−1^ of the redox couple Fe(CN)_6_]^3−/4−^. CV scans were recorded in the potential range of −0.5 to 1.0 V vs. Ag/AgCl, with a scan rate of 50 mV s^−1^. An open circuit potential (OCP) setup was employed for EIS measurements. The experimental conditions of EIS were: 10 points per decade, frequency range of 10^7^ and 10^−2^ Hz and amplitude of 10 mV. Equivalent circuit and fitting results were applied and obtained using the Electrochemical Circle Fit tool. The charge transfer resistance (R_ct_) and other components of the adjusted electrical circuit obtained during the analysis were used to obtain the quantitative signal of the RBD peak protein concentration in the assay.

### 2.6. Analysis of Spike Protein RBD in Saliva Samples

The saliva analysis was conducted by diluting the sputum-collected sample with PBS 7.4 (1:1) prior to incubation. Sputum samples from the oral cavity were collected on an empty stomach and before morning oral hygiene to avoid contamination by toothpaste and remnants of food or coffee. No complementary extraction or purification procedures were employed. In order to evaluate the immunoassay response, electrodes were incubated with only PBS, sample and PBS (1:1) and sample and PBS (1:1) contaminated with 2.5 μg/mL of spike protein RBD. Then, the immunosensor was rinsed carefully with PBS and the electrochemical measurements were recorded.

## 3. Results and Discussion

### 3.1. Morphological and Electrochemical Characterization of the rGO and the SARS-CoV-2 Immunosensor

**Figure 1:**
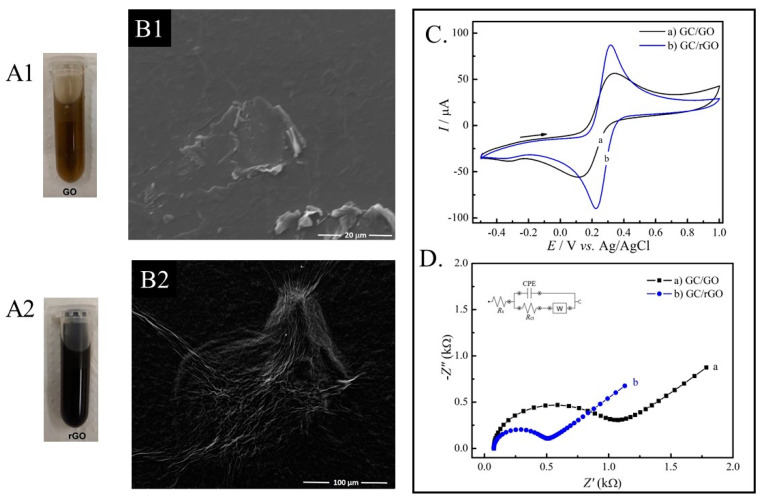
Visual characterization of GO (**A1**) and rGO (**A2**) suspensions. SEM images of GO (**B1**) and rGO (**B2**). Electrochemical characterization of electrodes modified with GO and rGO materials using CV (**C**) and EIS (**D**).

Before preparing the immunosensor, the rGO was morphologically, electrochemically and visually characterized, as shown in Figure 1. Different colors can be observed for the GO and rGO suspensions. The characteristic color of the GO suspension is yellowish, while after chemical reduction, the GO presents a darker color. This is a way to visually characterize the structural changes of the graphene [20]. In the microscopic analysis, it can be seen that the GO material consists of a mixture of single layers and multilayer graphene sheets and the rGO displays a wrinkled structure with plenty of defects and corrugations. The electrochemical characterization performed by CV shows the voltammetric profiles with well-defined oxidation and reduction peaks for the GO and rGO modified the GC electrodes. This behavior is due to the Fe^3+^/Fe^2+^ redox couple. The GC/rGO electrode showed a 1.6-fold increase in the peak current compared to the GC/GO electrode. This increase is due to the presence of defects introduced in its structure, as wellas fewer oxygen atoms increasing the electron transport [21]. In accordance with the CV experiments, the study by EIS showed a lower value of R_ct_ to rGO, indicating the improvement in the electronic transfer of this material. Therefore, the rGO obtained by the chemical method was successfully characterized showing that GO was reduced.

**Figure 2:**
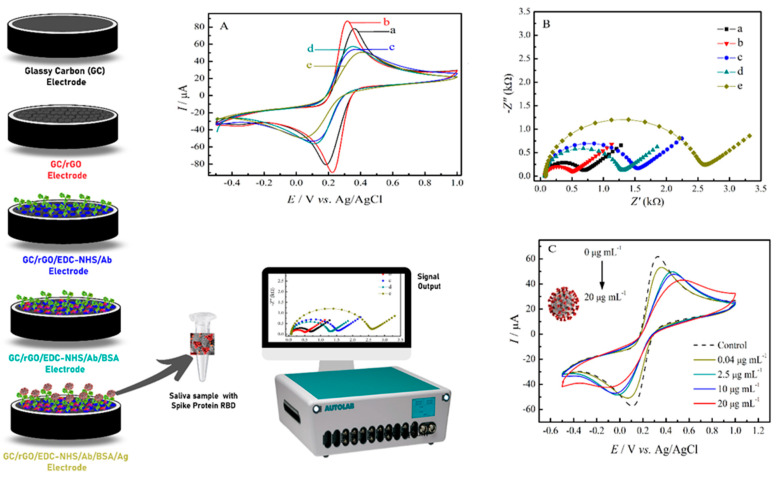
CV (**A**) and EIS (**B**) electrochemical characterization of the immunosensor steps fabrication in 0.2 mol L^−1^ PBS pH 7.4, 0.1 mol L^−1^ KCl containing 5.0 mmol L^−1^ of [Fe(CN_6_)]^3−^/^4−^ for the electrodes: (a) GC, (b) GC/rGO, (c) GC/rGO-EDC-NHS/Ab, (d) GC/rGO-EDC-NHS/Ab/BSA and (e) GC/rGO-EDC-NHS/Ab/BSA/Ag. (**C**) CV experiments in for the GC/rGO-EDC-NHS/Ab/BSA electrode (control) and after the incubation of different antigen concentration.

EIS and CV experiments were also used to monitor the single steps of the SARS-CoV-2 immunosensor assembly process as presented in Figure 2. The cyclic voltammograms obtained for the GC and GC/rGO electrodes showed well-defined oxidation and reduction peaks due to the Fe^3+^/Fe^2+^ redox couple. Hence, an average increase of 11.5% in the peak currents were observed when comparing the GC/rGO electrode (curve b) with the bare GC electrode (curve a). This increase can be attributed to the high electron transfer properties of chemically reduced graphene. As the immunosensor fabrication was carried on, with the steps of antibody immobilization (curve c), followed by BSA surface blocking (curve d) and then the SARS-CoV-2 spike protein RBD (antigen) incubation step (curve e), a decrease in the anodic and cathodic peak currents of the redox couple was observed. This occurs because the biomolecules act as an obstacle to the electron transfer at the electrode-solution interface. These results indicate that the SARS-CoV-2 antibody and antigen are bonded to the electrode surface. Moreover, ΔE_p_ of 237 mV for the GC/rGO-EDC-NHS/Ab electrode and 358 mV after the SARS-CoV-2 antigen is incubated are observed. This increase in the ΔE_p_ was also observed in the antigen-antibody binding procedures of different types of electrochemical immunosensors [19,22]. In addition, Figure 2C showed that the decrease of currents generated by the [Fe(CN)_6_]^4−^/^3−^ system observed in the cyclic voltammograms after SARS-CoV-2 antigen binding at the GC/rGO-EDC-NHS/Ab/BSA electrode has a clear correlation with the concentration of the antigen.

Figure 2B also presented the Nyquist plots for bare GC (■), GC/rGO (▼), GC/rGO-EDC-NHS/Ab (●), GC/rGO-EDC-NHS/Ab/BSA (▲) and GC/rGO-EDC-NHS/Ab/BSA/Ag (♦) electrodes. The EIS results were quantitatively optimized in a Randles equivalent circuit (inset Figure 2B) in order to calculate the charge-transfer resistance (R_ct_), the electrolyte ohmic resistance (R_s_), the constant phase element (CPE) and the surface roughness (α). The EIS experimental values obtained are summarized in Table 1. As expected and in agreement with CV experiments, a lower value of R_ct_ for rGO was observed, indicating the improvement in electron transfer of when the GC electrode is modified with this material. However, when biomolecules, such as proteins and enzymes, that have poor electrical conductivity at low frequencies are immobilized on the electrodes surface, the electron transfer process between the solution-based mediators and the electrode surface is impeded. Thus, an R_ct_ value of 1464.5 Ω for the GC/rGO-EDC-NHS/Ab electrode (curve c) was found, and after the SARS-CoV-2 antigen binding at the GC/rGO-EDC-NHS/Ab/BSA electrode (curve e), the R_ct_ value was 2398.8 Ω. This behavior of increase in R_ct_ as the deposition of the biomaterial occurs on the biosensor surface is reported in several studies [23,24]. Leva-Bueno et al. [25] did a general scheme of EIS for each step of biosensor construction, showing that the impedance increases as the deposition over the surface electrode increases. Therefore, the CV and EIS experiments indicated that the SARS-CoV-2 immunosensor was effectively prepared.

### 3.2. Optimization and Stability of the Impedimetric Immunosensor for SARS-CoV-2 Spike Protein RBD

**Figure 3:**
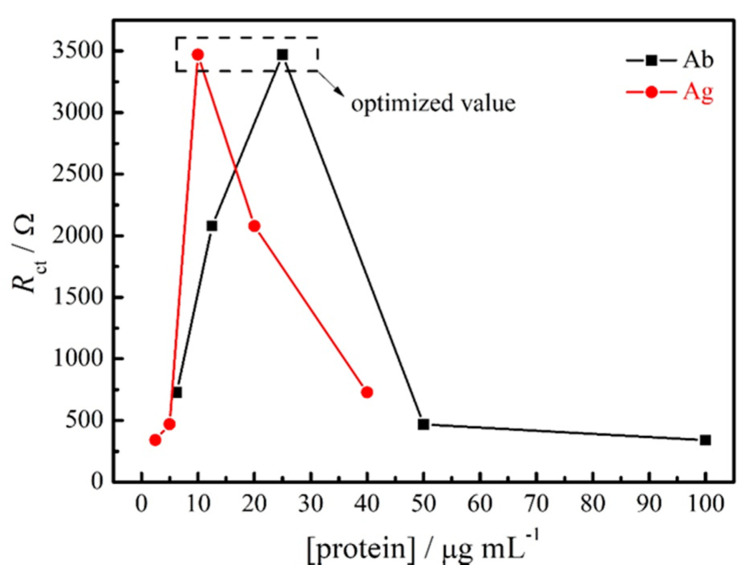
Optimization of antibody and antigen proteins concentrations at the immunosensor surface by EIS experiments.

The optimization of the impedimetric immunosensor was carried out with the antibody and antigen aliquots diluted from stock solutions with filtered PBS pH 7.4. The optimization experiments were conducted by diluting the antibody (Ab) at 1:1600, 1:800, 1:400, 1:200 and 1:100 (stock solution: 1.0 mg mL^−1^), and corresponding antigen (Ag) dilutions at 1:10, 1:20, 1:40, 1:80 and 1:160 (stock solution: 0.2 mg mL^−1^). The results presented in Table 2 and Figure 3 represent the effect of Ab and Ag concentration on the R_ct_ value during the immunoassay. It is possible to observe that the highest increase in the R_ct_ value was obtained for 1:40 Ab and 1:20 Ag dilutions. Therefore, this proportion was considered as an optimal value and used in the next studies.

To evaluate the stability of the proposed immunosensor, EIS and CV experiments were performed for the GC/rGO-EDC-NHS/Ab and GC/rGO-EDC-NHS/Ab/BSA/Ag electrodes. Five sequential experiments were carried out for the GC/rGO-EDC-NHS/Ab electrode, and it is observed that the I_pa_/I_pc_ and R_ct_ values did not show significant differences between the measurements presenting an average value of 1.44 ± 0.03 µA (*n* = 5) and 1350.02 ± 70.60 Ω (*n* = 5), respectively, thus demonstrating that antibody proteins were effectively immobilized on the GC/rGO-EDC-NHS surface (Figure not shown).

### 3.3. Analytical Performance of the SARS-CoV-2 Immunosensor

**Figure 4:**
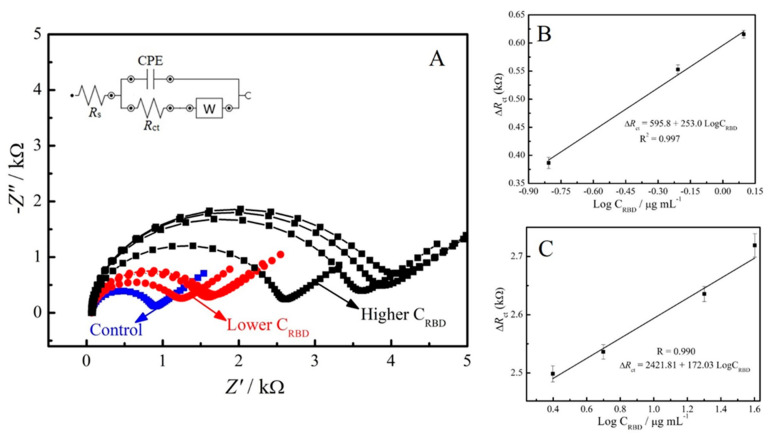
(**A**) EIS responses of the impedimetric immunosensor with different concentrations of the SARS-CoV-2 spike protein. The respective calibration curves plotted between the ΔR_ct_ and logarithmic concentration of SARS-CoV-2 spike protein from (**B**) 0.16 to 1.25 μg/mL, and (**C**) 2.5 to 40 μg/mL.

The analytical performance of SARS-CoV-2 was evaluated by using the Nyquist plots obtained from the EIS experiments at different concentrations of SARS-CoV-2 spike protein RBD. As shown in Figure 4, R_ct_ values were enhanced with the increase of the antigen concentration, indicating a clear dependence on target concentration. The resulting calibration plots presented a good liner relationship between ΔR_ct_ (subtraction of electrode’s R_ct_ before and after spike protein RBD incubation) and the logarithm concentrations of the antigen. In addition, two linear segments were obtained with different slopes. The first segment of the analytical curve is linear for a protein concentration range of 0.16 to 1.25 μg/mL (●). Meanwhile, the second segment of the calibration curve is also linear for a range of 2.5 to 40 μg/mL (■) RBD S protein concentration. The detection limit (calculated as LOD = 3SDblank/Slope) obtained for the lowest antigen concentrations was 150 ng/mL.

**Figure 5:**
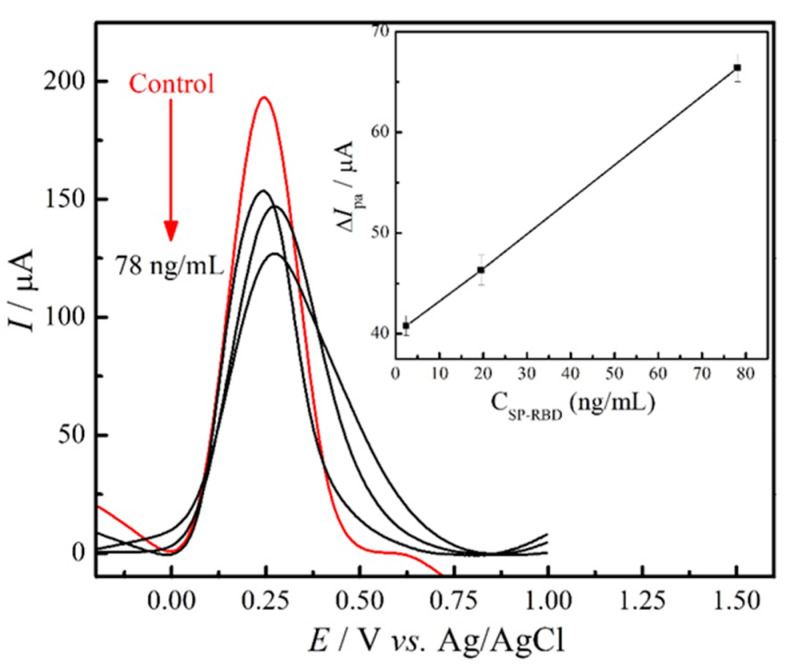
SWV data of the immunosensor in the absence (control) and presence of spike protein RBD concentrations ranging from 2.44 to 78 ng/mL; the inset shows the relationship between the Δ*I*_pa_ and concentration of the SARS-CoV-2 spike protein.

The sensitivity of the analytical device is a crucial point for the detection of the disease at the beginning of the infection. It is known that PCR test, mainly in saliva samples, does not detect the virus in the first days of the infection. Therefore, low cost and high sensitivity analytical methods are very important. The diagnostic platform developed in this work can be used for SARS-CoV-2 detection using other voltammetric techniques, such as square wave voltammetry (SWV), which increases the sensitivity of the proposed diagnostic. Figure 5 shows the square wave voltammograms obtained for the control (GC/rGO-EDC-NHS/Ab/BSA electrode) and after the binding of different concentrations of SARS-CoV-2 antigen. Using the SWV technique, the proposed sensor detected a concentration of 2.40 ng/mL of the virus. This study shows the potential of the technique and the immunosensor proposed in the SARS-CoV-2 spike protein analysis at low concentrations [26,27,28].

### 3.4. SARS-CoV-2 Spike Protein RBD Analysis in Saliva Samples

**Figure 6:**
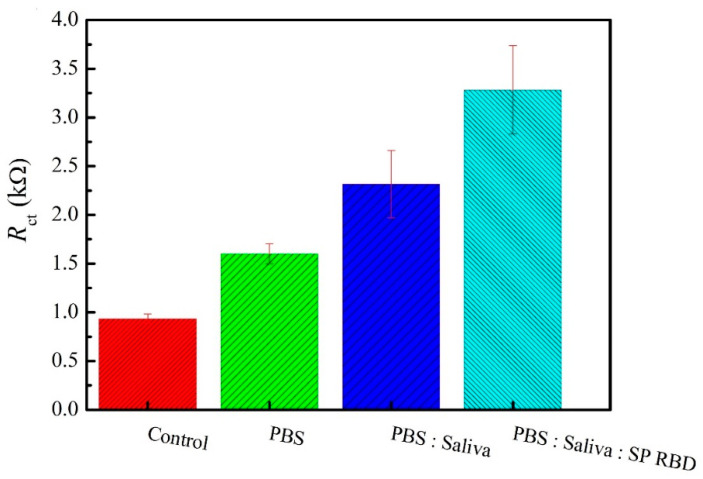
Response of the proposed immunosensor towards detecting SARS-CoV-2 spike protein RBD in real saliva sample.

The analysis of the SARS-CoV-2 spike protein RBD in saliva samples was performed by EIS experiments in triplicate. The samples preparation is described in Section 2.6 and the obtained results are presented in Figure 6. As expected, it is possible to observe the increase in the R_ct_ values when the saliva samples were spiked with virus. The mean R_ct_ values found in the presence of 2.5 μg/mL of SARS-CoV-2 spike protein RBD were 3283.2 ± 451.5 Ω (*n* = 3), and in the absence of the antigen were 2316.2 ± 345.1 Ω (*n* = 3). The immunosensor showed a good response towards SARS-CoV-2 determination in the saliva samples. The proposed immunosensor is an effective tool towards early COVID-19 diagnosis. The European Union (EU) has stated that antibody-based kits have limitations in detecting SARS-CoV-2 infections because antibodies only became detectable within several days after infection [29,30]. In addition, the saliva samples are much easier and less invasive method, and depending on age, it can be done even by self-collection [31,32,33].

## 4. Conclusions

A new methodology based on an electrochemical immunosensor developed with reduced graphene oxide for SARS-CoV-2 determination was successfully described in this work, presenting a low-cost technology by employing glassy carbon electrodes modified with rGO, a graphene material derivative through electrochemical reduction, which has an inexpensive, easy, fast and green way of obtention if compared with other materials also employed as biosensor surface modifiers, such as gold and silver. The large surface area of this material allows the coupling of interested biomolecules, and its conductivity properties can be enhanced, functionalizing it with immobilization agents, as EDC/NHS, and this type of surface modification can easily be transposed to printed carbon electrodes, which enables the integration of this immunosensor in point-of-care (POC) devices.

The immunosensor was characterized and optimized by electrochemical techniques and successfully applied to the determination of the SARS-CoV-2 spike protein RBD in saliva samples. Compared with other diagnostic methods and developed biosensors aiming the detection of SARS-CoV-2, this work combines feasibility and reliability, without any complex steps of building it, and is less reactive and time consuming compared to RT-PCR, having a great potential for large-scale production of a diagnostic tool with medical care capability and not needing specialized personnel in its management, contributing to a more effective control of the spread of SARS-CoV-2. In addition, the immunosensor demonstrated robustness towards SARS-CoV-2 analysis, showing good reproducibility and contributing to advances in the SARS-CoV-2 electrochemical biosensing area.

## Figures and Tables

**Table 1:** Resume of fitted parameters of EIS experiments.

Steps of Immunosensor Fabrication	R_ct_ (Ω)	R_s_ (Ω)	CPE (µF sα^−1^)	α
GC	720	69.5	1.49	0.95
GC/rGO	550	91	1.30	0.97
GC/rGO-EDC-NHS/Ab	1464.5	67.6	0.79	0.97
GC/rGO-EDC-NHS/Ab/BSA	1241.7	60	0.81	0.94
GC/rGO-EDC-NHS/Ab/BSA/Ag	2398.8	81.7	0.75	1.00

**Table 2:** Effect of antibody and antigen dilution on the immunosensor response.

Dilution		R_ct_ (Ω)
Ab	Ag	
1:1600	1:10	727.81
1:800	1:20	2078.5
1:400	1:40	3470.4
1:200	1:80	468.63
1:100	1:160	340.36

## Data Availability

The data is available under the request to the correspondence.
